# Determinants of Aortic Stiffness: 16-Year Follow-Up of the Whitehall II Study

**DOI:** 10.1371/journal.pone.0037165

**Published:** 2012-05-22

**Authors:** Nanna B. Johansen, Dorte Vistisen, Eric J. Brunner, Adam G. Tabák, Martin J. Shipley, Ian B. Wilkinson, Carmel M. McEniery, Michael Roden, Christian Herder, Mika Kivimäki, Daniel R. Witte

**Affiliations:** 1 Steno Diabetes Center A/S, Gentofte, Denmark; 2 Department of Epidemiology and Public Health, University College London, London, United Kingdom; 3 Semmelweis University Faculty of Medicine, 1st Department of Medicine, Semmelweis University, Budapest, Hungary; 4 Clinical Pharmacology Unit, University of Cambridge, Addenbrooke's Hospital, Cambridge, United Kingdom; 5 Institute for Clinical Diabetology, German Diabetes Center, Leibniz Center for Diabetes Research at Heinrich Heine University Düsseldorf, Düsseldorf, Germany; 6 Department of Metabolic Diseases, Heinrich Heine University Düsseldorf, Düsseldorf, Germany; Lausanne University Hospital and University of Lausanne, Switzerland

## Abstract

**Background:**

Aortic stiffness is a strong predictor of cardiovascular disease endpoints. Cross-sectional studies have shown associations of various cardiovascular risk factors with aortic pulse wave velocity, a measure of aortic stiffness, but the long-term impact of these factors on aortic stiffness is unknown.

**Methods:**

In 3,769 men and women from the Whitehall II cohort, a wide range of traditional and novel cardiovascular risk factors were determined at baseline (1991–1993) and aortic pulse wave velocity was measured at follow-up (2007–2009). The prospective associations between each baseline risk factor and aortic pulse wave velocity at follow-up were assessed through sex stratified linear regression analysis adjusted for relevant confounders. Missing data on baseline determinants were imputed using the Multivariate Imputation by Chained Equations.

**Results:**

Among men, the strongest predictors were waist circumference, waist-hip ratio, heart rate and interleukin 1 receptor antagonist, and among women, adiponectin, triglycerides, pulse pressure and waist-hip ratio. The impact of 10 centimeter increase in waist circumference on aortic pulse wave velocity was twice as large for men compared with women (men: 0.40 m/s (95%-CI: 0.24;0.56); women: 0.17 m/s (95%-CI: −0.01;0.35)), whereas the opposite was true for the impact of a two-fold increase in adiponectin (men: −0.30 m/s (95%-CI: −0.51;−0.10); women: 0.61 m/s (95%-CI: −0.86;−0.35)).

**Conclusion:**

In this large prospective study, central obesity was a strong predictor of aortic stiffness. Additionally, heart rate in men and adiponectin in women predicted aortic pulse wave velocity suggesting that strategies to prevent aortic stiffening should be focused differently by sex.

## Introduction

Aortic pulse wave velocity (aPWV), a measure of aortic stiffness, is a robust predictor of cardiovascular disease in the general population and in high risk populations as shown in a recent metanalysis [1]. This predictive association is independent of traditional risk factors, such as blood pressure, lipids, and smoking, and extends over and above the effect of other indicators of arterial stiffness, such as brachial pulse pressure, central pulse pressure and carotid-brachial pulse pressure amplification [2]. Thus, aPWV has been used as an intermediate outcome in several randomized trials to determine cardiovascular effects of antihypertensive treatment [3], lipid-lowering treatment [4], and weight loss interventions [5].

The concept that aPWV is both an independent risk factor and an intermediate marker of cumulated cardiovascular disease risk is increasingly accepted [6] and is supported by a recent review [7] of several large-scale studies confirming the cross-sectional association between cardiovascular risk factors and aPWV. However, current knowledge is limited on the prospective association between cardiovascular risk factors and aPWV [8], [9]. In the Caerphilly study [9] including men only, heavy smoking, C-reactive protein (CRP) and pulse pressure were strong predictors of aPWV 20 years later, but the predictive value of other biomarkers remains unclear. In cross-sectional studies, central obesity and inflammation have been strongly associated with aortic stiffness [10]–[12]. It is unknown, however, whether waist circumference and a wide range of inflammatory markers can also predict aortic stiffness in follow-up examinations. Given the importance of identifying early determinants of aortic stiffness, prospective investigations of these associations in men and women throughout the normal and moderately elevated ranges of cardiovascular risk factors seem warranted.

In this study from the Whitehall II cohort of British middle-aged men and women, we sought to examine the extent to which a wide range of cardiovascular risk factors are associated prospectively with aPWV.

## Methods

### Study population

The Whitehall II Study is an occupational cohort including 10,308 British civil servants aged 35–55 at study inception in 1985. The cohort has been followed with clinical examinations every 5 years and additionally with questionnaires every 2–3 years up to the end of 2009 (phase 9). Details of the study have been provided [13]. Only the 9,181 participants of White ethnicity are used in this study (89%).

The phase 3 examination in 1991–1993 is the baseline for the present study, as this was the first time a wide range of cardiovascular risk factors were measured. Measurement of some of these risk factors was repeated in the 2007–2009 clinical examination (phase 9) when also the first assessment of aPWV was performed. A total of 7,955 (87%) participants attended the phase 3 clinical examination. At the phase 9 follow-up examination, 588 (7%) of these had died and another 1,347 (17%) chose not to participate. Of the 6,020 participants at phase 9, approximately two thirds had an aPWV measurement. For 60% of participants with no aPWV measurement, the reason for missing data on aPWV was station closure due to insufficient staff resources; 5% had atrial fibrillation and for 20% the carotid or femoral pulse could not be found. From the 3,894 participants with an aPWV measurement, we excluded those with previous non-fatal coronary heart disease at phase 3 (n = 81, 2%). To reduce any bias related to treated diabetes and inflammatory diseases, we further excluded subjects with known diabetes at phase 3 (n = 24, <1%) and those reporting use of systemic corticosteroids at any time up to phase 9 (n = 20, <1%), leaving 3,769 participants for analysis.

The University College London ethics committee reviewed and approved the study, and written informed consent was obtained from each participant at each examination phase. The study was conducted according to the principles of the Helsinki Declaration.

### Measurements at baseline

The measurements at baseline are described in further details in the Methods S1. Briefly, height, weight, waist- and hip circumference, blood pressure, and heart rate were measured according to a standard protocol. Venous blood samples were collected after an overnight fast in the morning or in the afternoon after no more than a light fat-free breakfast eaten before 08.00 h. After the initial venous blood samples were taken, the participants underwent a standard 2-hour glucose tolerance test. Plasma glucose and serum insulin were analyzed in both the fasting and 2-hour samples. In the fasting samples, total cholesterol, high density lipoprotein (HDL) cholesterol, low density lipoprotein (LDL) cholesterol (Friedewald equation), triglycerides, apolipoprotein A-I and B, lipoprotein (a), adiponectin, high sensitive CRP, interleukin 6 (IL-6), interleukin 1 receptor antagonist (IL-1Ra), fibrinogen, von Willebrand factor, factor VII activity, and β-carotene were analyzed.

Information on ethnicity, employment grade, smoking habits, alcohol consumption and physical activity were collected using a self-administered questionnaire.

We used the Homeostasis Model Assessment calculator version 2.2 [14] to calculate insulin resistance (HOMA2-IR) and β-cell function (HOMA2-%B) from the levels of fasting plasma glucose and fasting serum insulin. Insulin sensitivity was calculated from fasting- and 2-hour values of plasma glucose and serum insulin using the insulin sensitivity index (ISI_0–120_) [15].

### Measurements at follow-up

With the participant in a supine position, blood pressure was measured after 10 minutes of rest. From the supine systolic blood pressure and diastolic blood pressure, mean arterial pressure was calculated. The aPWV was then assessed between the carotid and femoral sites using applanation tonometry (SphygmoCor, Atcor Medical, Australia), which is a validated method of measuring aPWV [16]. The path length was determined with a tape measure by subtracting the carotid-sternal notch distance from the femoral-sternal notch distance. In each participant, aPWV was measured twice. If the difference in aPWV between the two measurements was larger than 0.5 m/s, a third measurement was taken. In the analyses, the average of the two closest measurements was used. In 125 of the participants, aPWV measurements were repeated within 60 days to assess the short term reproducibility. The median intra-individual difference in aPWV was 0.08 m/s (interquartile range: −0.68 to 0.93 m/s) [17].

Use of antihypertensive-, lipid-lowering-, and glucose-lowering medication was assessed throughout the follow-up period. We defined medication history as any known medication up to and including phase 9 and classified missing information as no known medication history.

Incident diabetes was assessed throughout the follow-up period and was based on a standard oral glucose tolerance test at the clinical examinations (phases 5, 7, and 9) according to the World Health Organisation definition [18]. Additionally, self-reports of diabetes or the use of glucose-lowering medication (phases 4, 5, 6, 7, 8 and 9) classified the participants with incident diabetes. The incidence of non-fatal coronary heart disease was assessed up to September 2004 [19].

### Statistical analysis

The following baseline determinants were considered separately in the analysis: waist- and hip circumference, waist-hip ratio, height, diastolic blood pressure, systolic blood pressure, pulse pressure, heart rate, total cholesterol, HDL cholesterol, LDL cholesterol, triglycerides, apolipoprotein A-I, apolipoprotein B, lipoprotein (a), adiponectin, CRP, IL-6, IL-1Ra, fibrinogen, von Willebrand factor, factor VII, β-carotene, alcohol intake (units/week), hours/week of vigorous exercise, fasting plasma glucose, 2-hour plasma glucose, HOMA2-%B, HOMA2-IR, and ISI_0–120_.

Baseline values of plasma glucose and serum insulin for participants who had been fasting for less than five hours (n = 322, 9%) were assigned as missing data. Prior to analysis, we removed outliers from all predictors and log-transformed predictors with a highly skewed distribution (adiponectin, CRP, IL-6 and IL-1Ra).

For most determinants around five percent or less of the values were missing. For plasma glucose, serum insulin and the major part of the inflammatory markers the proportions of missing values were slightly higher (7–18%). In the study population, a quarter of the data on β-carotene and half of the data on adiponectin and IL-1Ra were missing (Table S1). Missing data on baseline determinants were imputed using the Multivariate Imputation by Chained Equations (MICE) method in R software [20] with missing-at-random assumptions. Twenty copies of the data [21], each with missing values suitably imputed, were independently assessed in the analyses described below. Estimates of parameters of interest were averaged across the copies to give a single mean estimate. Standard errors and p-values were adjusted according to Rubin's rules [22].

The prospective associations between determinants at baseline and aPWV at follow-up were assessed through linear regression analysis stratified by sex and adjusted for mean arterial pressure at the time of the aPWV measurement [23]. We explored different levels of adjustment for potential confounders in the analyses; as a first step we adjusted for age and quadratic age. Secondly, analyses were additionally adjusted for BMI and lastly we further adjusted for smoking habits and employment grade as a measure of socioeconomic status. In all of the analyses of hemodynamic markers (diastolic blood pressure, systolic blood pressure, pulse pressure, heart rate) we also adjusted for history of anti-hypertensive treatment and for incident coronary heart disease. In the same manner, analyses of lipids (total cholesterol, HDL cholesterol, LDL cholesterol, triglycerides, apolipoprotein A-I, apolipoprotein B, lipoprotein (a)) were also adjusted for lipid-lowering treatment and analyses of indices of glucose metabolism (fasting plasma glucose, 2-hour plasma glucose, HOMA2-%B, HOMA2-IR, ISI_0–120_) were also adjusted for incident diabetes. Both standardized (per 1 standard deviation difference in the determinant) and non-standardized regression coefficients are presented.

A subset of the determinants was also measured at follow-up. The corresponding cross-sectional associations between these determinants and aPWV are given in Figure S1 and Table S2.

Analyses were performed using SAS version 9.2 (SAS Institute, Cary, NC, USA) and R version 2.13.0.

## Results

Participants were mainly men (76%). Women had lower blood pressure levels and fewer coronary heart disease events than men. Women had a more favorable lipid profile, and a lower proportion of women were on lipid-lowering or anti-hypertensive treatment (Table 1). Median follow-up time was 16.3 years (range: 13.1–17.6). Participants lost to follow-up and phase 9 participants with no aPWV measurement were more likely to be women, to be smokers and to be in lower employment grade, they were older and slightly more obese and with higher levels of low-grade inflammation compared to the study participants. This was in particular true for those lost to follow-up (Table S3).

**Table 1:** Characteristics of the study population at baseline and at follow-up.

	Men	Women
N	2,857	912
Baseline		
Age (years)	48.4 (5.7)	48.6 (5.9)
BMI (kg/m2)	24.8 (2.9)	24.6 (4.0)
Waist circumference (cm)	87.3 (8.6)	73.8 (10.6)
Hip circumference (cm)	96.6 (5.6)	95.9 (8.5)
Waist-hip ratio	0.90 (0.06)	0.77 (0.06)
Height (cm)	177.0 (6.4)	163.1 (6.2)
Diastolic blood pressure (mmHg)	80.2 (8.8)	75.3 (8.9)
Systolic blood pressure (mmHg)	120.7 (12.5)	115.3 (13.2)
Pulse pressure (mmHg)	40.4 (8.4)	40.0 (8.4)
Heart rate (bpm)	63.0 (10.5)	65.6 (9.8)
Total cholesterol (mmol/l)	6.4 (1.1)	6.3 (1.1)
HDL cholesterol (mmol/l)	1.3 (0.3)	1.7 (0.4)
LDL cholesterol (mmol/l)	4.4 (1.0)	4.1 (1.0)
Triglycerides (mmol/l)	1.5 (0.9)	1.0 (0.6)
Apolipoprotein A–I (mg/dl)	2.1 (0.3)	2.4 (0.4)
Apolipoprotein B (mg/dl)	1.3 (0.3)	1.2 (0.3)
Lipoprotein (a) (mg/dl)	29.9 (29.0)	31.0 (29.6)
Adiponectin (µg/ml)	7.8 (6.0;10.4)	13.1 (9.5;17.4)
CRP (mg/l)	0.7 (0.4;1.4)	0.8 (0.4;1.8)
IL-6 (pg/ml)	1.3 (0.9;1.8)	1.4 (1.0;2.1)
IL-1Ra (pg/ml)	234.9 (187.3;297.7)	259.2 (197.2;353.5)
Fibrinogen (g/l)	2.3 (0.5)	2.5 (0.6)
Von Willebrand factor (IU/dl)	102.3 (36.5)	101.9 (35.2)
Factor VII (% standard)	87.4 (21.6)	89.1 (23.2)
β-carotene (µmol/l)	0.9 (0.5)	1.0 (0.6)
Alcohol intake (units/week)	12.7 (13.6)	6.5 (7.2)
Vigorous exercise (hrs/week)	1.0 (1.6)	0.5 (1.2)
Employment grade (%)		
Administrative	44.1 (42.3;45.9)	20.5 (17.9;23.3)
Professional/executive	52.4 (50.5;54.2)	51.1 (47.8;54.4)
Clerical/support	3.5 (2.9;4.3)	28.4 (25.5;31.4)
Smoking habits (%)		
Never-smoker	50.3 (48.4;52.1)	55.2 (51.9;58.4)
Ex-smoker	37.2 (35.4;39.0)	28.9 (26.0;32.0)
Current smoker	8.8 (7.8;9.9)	11.8 (9.8;14.1)
Fasting plasma glucose (mmol/l)	5.3 (0.5)	5.0 (0.5)
2-hour plasma glucose (mmol/l)	5.4 (1.7)	5.6 (1.8)
HOMA2-%B (%)	77.8 (26.3)	81.1 (26.6)
HOMA2-IR	0.92 (0.48)	0.88 (0.49)
ISI_0,120_	45.0 (19.2)	41.5 (16.3)
Follow-up		
Aortic pulse wave velocity (m/s)	8.5 (2.0)	8.1 (1.9)
Mean arterial pressure (mmHg)	90.5 (10.3)	86.9 (11.3)
Diabetes incidence (%)	11.4 (10.2;12.6)	10.6 (8.7;12.8)
Non-fatal CHD incidence (%)	5.3 (4.5;6.1)	3.2 (2.1;4.5)
Anti-hypertensive treatment in history (%)	33.8 (32.0;35.5)	28.7 (25.8;31.8)
Lipid-lowering treatment in history (%)	31.5 (29.8;33.3)	23.4 (20.6;26.2)

Data are means (SD), medians (interquartile range) or proportions (95% CI) except for the number of participants (N).

BMI = body mass index; HDL = high density lipoprotein; LDL = low density lipoprotein; CRP = C-reactive protein; IL-6 = interleukin 6; IL-1Ra = interleukin 1 receptor antagonist; HOMA2-%B = β-cell function; HOMA2-IR = insulin resistance; ISI_0–120_ = insulin sensitivity index; CHD = coronary heart disease.

**Figure 1:**
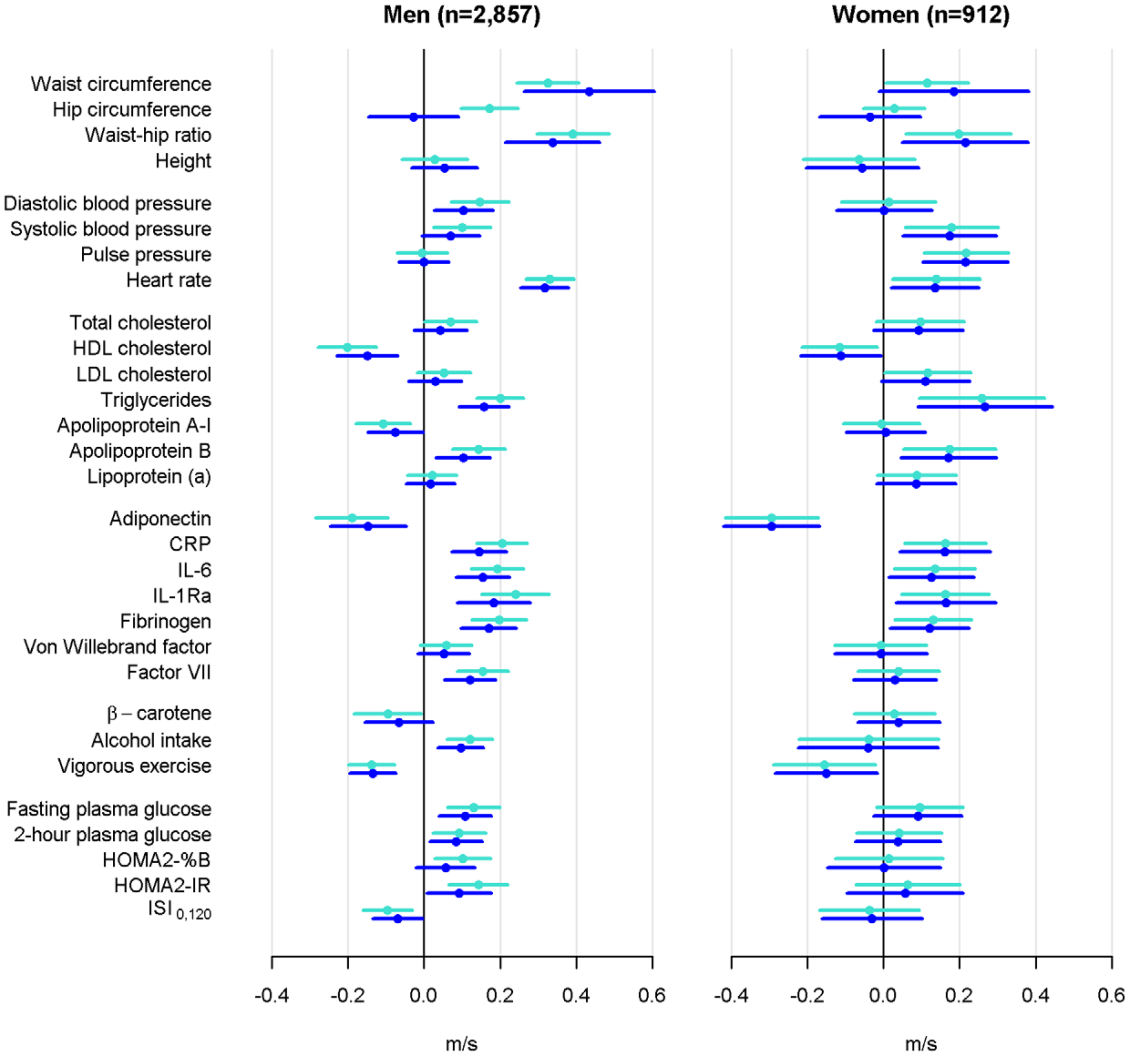
Standardized regression coefficients for predicting aortic pulse wave velocity. Light blue: adjustment for age, quadratic age, mean arterial pressure at the time of aortic pulse wave velocity measurement, and for relevant treatment and event history. Dark blue: further adjustment for body mass index. HDL = high density lipoprotein; LDL = low density lipoprotein; CRP = C-reactive protein; IL-6 = interleukin 6; IL-1Ra = interleukin 1 receptor antagonist; HOMA2-%B = β-cell function; HOMA2-IR = insulin resistance; ISI_0–120_ = insulin sensitivity index.

Central obesity (waist circumference and waist-hip ratio) was a strong predictor of aPWV 16 years later in both sexes, even after adjustment for BMI (Figure 1). Systolic blood pressure, heart rate, HDL cholesterol, triglycerides, apolipoprotein B, adiponectin, CRP, IL-1Ra, and fibrinogen were also significantly associated with aPWV in both sexes. Among men, waist circumference was the strongest determinant of aPWV at follow-up whereas among women triglycerides and adiponectin had the strongest association with aPWV (Figure 1).

For most determinants, adjustment for BMI slightly attenuated the association with aPWV in men, while the BMI adjustment had no effect in women. Indicators of central obesity remained strong predictors of aPWV and for both sexes waist circumference became a slightly stronger determinant for aPWV when adjusting for BMI (Figure 1). Further adjustment for employment grade and smoking habits had very little effect over adjustment for BMI and are therefore not shown.

The associations corresponding to the model adjusting for BMI are reported on the original scale of the determinants in Table 2. The impact of waist circumference on aPWV was twice as large for men compared with women, whereas the opposite was true for triglycerides and adiponectin. The effect of heart rate was almost three times as large for men, while pulse pressure was a strong predictor of aPWV among women only.

**Table 2:** Difference (95%-CI) in aortic pulse wave velocity at follow-up by a unit difference in baseline determinants.

Determinants	Men	Women
**Anthropometrics**		
Waist circumference (10 cm)	0.40 (0.24;0.56)‡	0.17 (−0.01;0.35)*
Hip circumference (10 cm)	−0.04 (−0.23;0.14)	−0.06 (−0.26;0.15)
Waist-hip ratio	4.12 (2.60;5.64)‡	2.63 (0.60;4.65)†
Height (10 cm)	0.06 (−0.04;0.16)	−0.06 (−0.23;0.11)
**Hemodynamic markers**		
Diastolic blood pressure (10 mmHg)	0.11 (0.03;0.20)†	0.00 (−0.14;0.14)
Systolic blood pressure (10 mmHg)	0.05 (0.00;0.11)*	0.14 (0.04;0.23)†
Pulse pressure (10 mmHg)∥	0.00 (−0.08;0.08)	0.26 (0.12;0.39)‡
Heart rate (10 bpm)∥	0.30 (0.24;0.37)‡	0.13 (0.02;0.24)*
**Lipids**		
Total cholesterol (mmol/l)	0.04 (−0.02;0.10)	0.08 (−0.02;0.19)
HDL cholesterol (mmol/l)	−0.38 (−0.58;−0.17)†	−0.28 (−0.55;−0.02)*
LDL cholesterol (mmol/l)	0.03 (−0.04;0.10)	0.11 (−0.01;0.23)*
Triglycerides (mmol/l)	0.18 (0.10;0.25)‡	0.30 (0.10;0.50)†
Apolipoprotein A–I (mg/dl)	−0.22 (−0.43;−0.01)*	0.02 (−0.28;0.32)
Apolipoprotein B (mg/dl)	0.36 (0.11;0.60)†	0.60 (0.16;1.03)†
Lipoprotein (a) (10 mg/dl)	0.01 (−0.02;0.03)	0.03 (−0.01;0.07)
**Inflammatory markers**		
Adiponectin (two-fold increase)	−0.30 (−0.51;−0.10)†	−0.61 (−0.86;−0.35)‡
CRP (two-fold increase)	0.13 (0.07;0.19)‡	0.14 (0.04;0.25)†
IL-6 (two-fold increase)	0.28 (0.15;0.40)‡	0.23 (0.02;0.43)
IL-1Ra (two-fold increase)	0.46 (0.22;0.70)†	0.42 (0.08;0.75)†
Fibrinogen (g/l)	0.33 (0.19;0.48)‡	0.24 (0.04;0.44)
Von Willebrand factor (10 IU/dl)	0.01 (0.00;0.03)	0.00 (−0.04;0.03)
Factor VII (10% standard)	0.05 (0.02;0.09)†	0.01 (−0.04;0.06)
**Lifestyle**		
β-carotene (mmol/l)	−0.13 (−0.31;0.05)	0.08 (−0.13;0.30)
Alcohol intake (10 units/week)	0.08 (0.03;0.12)†	−0.03 (−0.18;0.11)
Vigorous exercise (hrs/week)	−0.09 (−0.13;−0.05)‡	−0.10 (−0.19;−0.01)*
Ex-smoker vs. never smoker	−0.01 (−0.14;0.13)	−0.09 (−0.33;0.14)
Current smoker vs. never smoker	0.15 (−0.08;0.38)	−0.06 (−0.39;0.26)
**Glucose metabolism**		
Fasting plasma glucose (mmol/l)	0.21 (0.07;0.34)†	0.17 (−0.05;0.39)
2-hour plasma glucose (mmol/l)	0.05 (0.01;0.09)†	0.02 (−0.04;0.09)
HOMA2-%B (100 units)	0.20 (−0.08;0.48)	0.01 (−0.52;0.53)
HOMA2-IR	0.19 (0.01;0.37)*	0.12 (−0.20;0.44)
ISI_0,120_ (100 units)	−0.35 (−0.69;−0.02)*	−0.16 (−0.83;0.52)

Analyses were adjusted for age, quadratic age, BMI, mean arterial pressure at the time of aortic pulse wave velocity measurement, relevant treatment and events (Hemodynamic markers: anti-hypertensive treatment and coronary heart disease events; lipids: lipid-lowering treatment; glucose metabolism: diabetes incidence).

* P<0.05,

† P<0.01,

‡ P<0.0001 for significance of the determinant.

§ P<0.05,

∥ P<0.01 for sex difference in the determinant.

BMI = body mass index; HDL = high density lipoprotein; LDL = low density lipoprotein; CRP = C-reactive protein; IL-6 = interleukin 6; IL-1Ra = interleukin 1 receptor antagonist; HOMA2-%B = β-cell function; HOMA2-IR = insulin resistance; ISI_0–120_ = insulin sensitivity index; CHD = coronary heart disease.

Corresponding associations between risk factors and aPWV were seen in the cross-sectional analysis except that the associations with most of the indicators of glucose metabolism were stronger cross-sectionally, while the association with adiponectin was weaker. The absolute impact of heart rate on aPWV was higher in the cross-sectional analysis, especially for women (Figure S1 and Table S2).

## Discussion

In this study of 3,769 men and women of White ethnicity, we found that several traditional cardiovascular risk factors as well as indicators of low-grade inflammation were associated with aortic stiffness 16 years later, and that the pattern of associations differed by sex. The strongest determinants of aortic stiffness, in order of magnitude, were waist circumference, waist-hip ratio, heart rate and IL-1Ra among men, and adiponectin, triglycerides, pulse pressure and waist-hip ratio among women.

To our knowledge this is the first study to examine the long term effect of traditional and novel cardiovascular risk factors on aortic stiffness in both men and women. In our study, central obesity and low-grade inflammation were strong predictors of aortic stiffness in both sexes. A marked difference between sexes was observed in the impact of triglycerides and adiponectin, which was twice as high in women, and in heart rate which was almost three times as high in men than in women.

Our findings among men are broadly in line with those from the Caerphilly study [9] on 825 Welsh men during 20 years of follow-up. However, there were differences in the ranking of the determinants between the two studies, as low-grade inflammation was one of the strongest determinants of aortic stiffness and had a much larger impact than central obesity on aortic stiffness in the Caerphilly study. These differences may be attributable to the lower mean age and healthier risk profile of the men in the Whitehall II cohort and may also reflect the difference in level of adjustment in the analyses. Both studies adjusted for age and mean arterial pressure. We additionally adjusted for quadratic age, BMI and medication- and event history where relevant, whereas the Caerphilly study instead adjusted for heart rate and medication at time of follow-up only.

The observed sex differences in the relative importance of long-term determinants of aortic stiffness are important as it may point to differences in the etiology of cardiovascular disease between men and women. These findings also hold possible clues for how preventive strategies might be targeted differently by sex. It is, however, important to note that in the occupational Whitehall II cohort lower employment grades are overrepresented among women compared to men. This key indicator of socioeconomic status [24] has shown to be strongly related to cardiovascular risk factors and general health in this cohort [13]. Although we found no confounding effect of employment grade in our analyses, we cannot fully discount the role of the sex specific socio-economic structure in the Whitehall II population.

### Determinants of aortic stiffness

#### Central obesity and inflammation

Waist circumference and waist-hip ratio were among the strongest predictors of aortic stiffness among men and women, but with somewhat higher effect in men. The association with waist circumference was strengthened upon adjustment for BMI indicating that central obesity is the main contributing factor to the association between obesity and aortic stiffness. Our findings are in accordance with a cross-sectional study measuring visceral fat by computer tomography in 2,488 men and women. The study found that besides systolic blood pressure, visceral fat had the strongest independent association with aPWV [11]. The importance of fat accumulation as a determinant of aortic stiffness was further highlighted by our findings of a strong negative association between adiponectin and aortic stiffness in both sexes. Results from cross-sectional studies are all in line with our results [25], [26].

We also found other markers of low-grade inflammation, such as CRP, IL-6, IL-1Ra and fibrinogen to predict aortic stiffness in both sexes. Factor VII were additionally associated with aortic stiffness in men. These findings are in line with the Caerphilly study, which found an association of CRP and fibrinogen with aortic stiffness in men [9] and several cross-sectional studies reporting a strong association between CRP and aPWV [10], [12]. There are only few studies on the association between IL-6, IL-1Ra, fibrinogen, von Willebrand factor and factor VII and aPWV, and the results are inconsistent [10], [27], [28].

Although the association between inflammation and aortic stiffness may merely reflect the inflammatory burden caused by aortic stiffness or its determinants, an experimental study showed that acute systemic inflammation induced by vaccination with *Salmonella typhi* increases aPWV [29], supporting the hypothesis that inflammation is actually on the causal pathway leading to aortic stiffness. A further study supports this concept by showing that aortic stiffness is increased in people with rheumatoid arthritis, and that stiffness may be reversed by immunomodulatory therapy [30].

#### Lipids

Dyslipidaemia is an important, well-established and modifiable cardiovascular risk factor. However, apolipoprotein A–I and apolipoprotein B may have an even stronger effect on coronary heart disease [31]. We are the first to study the impact of a detailed lipid profile on aortic stiffness and found that HDL cholesterol and apolipoprotein B were associated with aortic stiffness in both sexes but with higher absolute and relative magnitude of apolipoprotein B in women (Table 2 and Figure 1). Additionally, LDL cholesterol in women and apolipoprotein A–I in men were to a lesser degree associated with aortic stiffness.

We found triglycerides to have a robust association with aortic stiffness in both sexes, but with an association twice as strong in women compared to men. This striking sex difference is also supported by a meta-analysis on incident coronary heart disease [32]. The mechanisms behind this sex difference are poorly understood but may be a result of sex-specific differences in lipid metabolism. Our findings are in line with the findings of the Framingham Offspring Study, which identified apolipoprotein B and HDL cholesterol as major determinants of incident coronary heart disease [33].

#### Glucose metabolism

Few studies exist on the long term effect of markers of glucose metabolism on aortic stiffness. In our study, the magnitude of the association between glucose metabolism and aortic stiffness after 16 years was comparable to that of inflammation and blood pressure in men but smaller than that of triglycerides and obesity. We found no association with aPWV in women. The Caerphilly study measured fasting plasma glucose as a marker of glucose metabolism but found no association with aortic stiffness.

#### Heart rate and blood pressure

There were also marked sex differences in the association between heart rate and pulse pressure and aortic stiffness. The association with heart rate was almost three times higher in men as in women, whereas pulse pressure was significantly associated with aortic stiffness in women only. The findings in the Caerphilly study on heart rate and pulse pressure are not in agreement with our results in men, which could be explained by the Whitehall II cohort being younger and thus pulse pressure may be a less accurate reflection of aortic stiffness.

#### Alcohol

The association between alcohol consumption and aortic stiffness differed by sex. Among men, we found a statistically significantly positive association with aortic stiffness. In women, however, the association was negative, although not statistically significant. This difference between sexes could be a reflection of social class, as the alcohol intake was markedly larger among highly educated women than among women with no education, whereas the difference in alcohol consumption across education level among men was less pronounced [34].

### Strengths and limitations

This study included middle-aged British civil servants of White ethnicity limiting the generalizability of our results. Due to the lack of a baseline measure of aPWV, we cannot conclude on causality. Clinical trials targeting the identified risk factors for aortic stiffness are needed to assess causality. A tape measure was used to determine the carotid-femoral path length which may have overestimated the distance in obese individuals resulting in an overestimated aPWV.

For most of the determinants 5% or more were missing. Instead of complete case analysis, we have used multiple imputation to handle the large number of missing data in this study. Several simulation studies have shown that complete case analysis generally leads to biased estimates, but that multiple imputation reduces this bias and increases precision [35], [36]. MICE is currently the state-of-the-art-method of dealing with data missing at random [37].

Of the 7,955 participants attending the phase 3 examination, 3,894 had measurements of aPWV at follow-up. We cannot exclude bias due to the healthy survivor effect, which might have weakened the associations, but the ranking of the determinants should not be affected by this effect.

We cannot fully conclude on whether the found associations are truly predictive or merely an effect of the risk tracking over time. We have adjusted the analyses for medication history, incident diabetes and coronary heart disease to account for temporal instability in the predictor variables that was outside the natural course of ageing, and the ranking and strength of the associations were largely replicated in the cross-sectional analyses. However, future studies assessing the trajectories of risk factors up to the time of aortic stiffness measurement would enable a more precise quantification of the impact of the risk factors over time.

In conclusion, this large prospective study of middle-aged men and women found central obesity and low-grade inflammation to be strong predictors of aPWV in both sexes. In addition, heart rate in men and adiponectin and triglycerides in women were strongly associated with aPWV suggesting that prevention strategies targeting aortic stiffness should focus on central obesity and heart rate among men and triglycerides and central obesity among women.

## Supporting Information

Figure S1 **Standardized cross-sectional regression coefficients for association with aortic pulse wave velocity.**
(DOC)

Methods S1 **Detailed information on baseline measurements.**
(DOC)

Table S1 **The fraction of the study participants with no information on the baseline determinant.**
(DOC)

Table S2 **Difference (95%-CI) in aortic pulse wave velocity at follow-up by a unit difference in determinants at follow-up.**
(DOC)

Table S3 **Baseline characteristics by follow-up participation and aortic pulse wave velocity measurement for those alive at follow-up.**
(DOC)
