# Supplementary material for: Determinants of Aortic Stiffness: 16-Year Follow-Up of the Whitehall II Study
Source: PLoS One. 2012 May 22;7(5):e37165. doi: 10.1371/journal.pone.0037165 (PMC3358295; doi:10.1371/journal.pone.0037165)
Supplement: Figure S1 — Standardized cross-sectional regression coefficients for association with aortic pulse wave velocity. (DOC) [file pone.0037165.s001.doc]

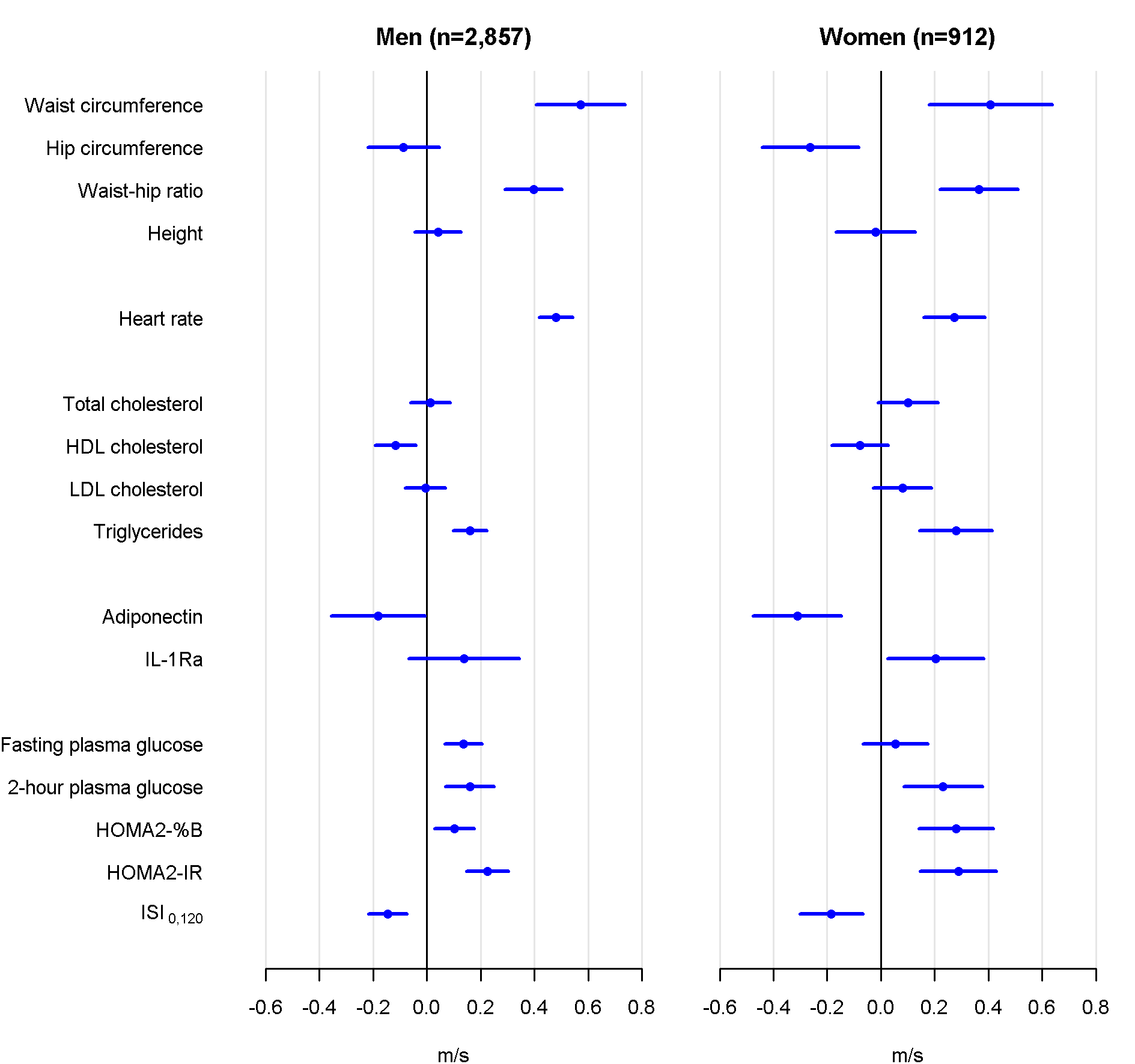


**Figure S1** Standardized cross-sectional regression coefficients for association with aortic pulse wave velocity.

The coefficients are adjusted for age, quadratic age, body mass index, mean arterial pressure at the time of aortic pulse wave velocity measurement and relevant treatment and event history.

HDL = high density lipoprotein; LDL = low density lipoprotein; IL-1Ra = interleukin 1 receptor antagonist; HOMA2-%B = β-cell function; HOMA2-IR = insulin resistance; ISI_0-120_ = insulin sensitivity index.
