# Supplementary material for: Determinants of Aortic Stiffness: 16-Year Follow-Up of the Whitehall II Study
Source: PLoS One. 2012 May 22;7(5):e37165. doi: 10.1371/journal.pone.0037165 (PMC3358295; doi:10.1371/journal.pone.0037165)
Supplement: Methods S1 — Detailed information on baseline measurements. (DOC) [file pone.0037165.s002.doc]

Methods S1

Measurements at baseline

The measurements at baseline have previously been described^1-5^. Height was measured to the nearest millimeter using a stadiometer with the participant standing erect with the head in the Frankfurt plane. Weight, waist- and hip circumference were measured with all items of clothing removed except for underwear. Weight was measured to the nearest 0.1 kg. Waist- and hip circumference were measured with the participant in a standing position utilizing a fiberglass tape-measure at 600 g tension. The waist circumference was taken as the smallest circumference at or below the costal margin and the mean of two waist measurements was used in the analyses. Hip circumference was measured at the level of the greater trochanter. Blood pressure was measured twice in a sitting position after five minutes rest by using a Hawksley random zero sphygmomanometer. The mean of the two values was used. Heart rate was assessed from the ECG recording.

Venous blood samples were collected after an overnight fast or in the afternoon after no more than a light fat-free breakfast eaten before 08.00 h (at least 5 hours of fasting). After the initial venous blood samples the participants drank 389 ml of Lucozade (equivalent to 75 g anhydrous glucose) over five minutes. A second venous blood sample was taken two hours later. Plasma glucose and serum insulin were analyzed in both the fasting and 2-hour samples. Glucose was determined in fluoride plasma by an electrochemical glucose oxidase method. Serum insulin was measured by radioimmunoassay using a polyclonal guinea-pig antiserum. In the fasting samples lipid profile, inflammatory markers and vitamins were analyzed. Cholesterol and triglycerides were measured in a centrifugal analyzer by enzymatic colorimetric methods. HDL cholesterol was determined after dextran sulphate-magnesium chloride precipitation of non-HDL cholesterol. LDL-cholesterol was derived from the Friedewald equation^6^. Apolipoprotein A-I and B were determined by an immunoturbidimetric method. C-reactive protein was measured using a high-sensitivity immunonephelometric assay, interleukin 6 was measured using a high-sensitivity ELISA assay, interleukin 1 receptor antagonist and adiponectin were measured with Quantikine ELISA kits (R&D Systems, Wiesbaden, Germany), fibrinogen was determined by an automated modification of the Clauss method, von Willebrand factor antigen was measured by double antibody ELISA and factor VII activity was measured by the Brozovic method. Plasma concentrations of β-carotene were determined by normal phase high performance liquid chromatography using a modification of the methods of Buttriss and Diplock^7^.

Smoking habits, alcohol consumption and hours of mild, moderate and vigorous physical activity were measured using a self-administered questionnaire^8^. Participants who reported smoking were defined as current smokers. Those who reported not smoking, and were not identified as current or ex-smokers in previous phases, were classified as never smokers. Ex-smokers were those participants who reported past smoking at phase 3 or current or ex-smoking at previous phases. Alcohol consumption, expressed in units, was based on the reported number of drinks (beer, wine, or spirits) the participants had consumed the last seven days. In the UK a standard measure of spirits, a glass of wine, and a half-pint of beer is considered to contain one unit (eight grams) of alcohol. Frequency and duration of mild, moderate, and vigorous physical activity was self-reported, and hours per week at each intensity level were calculated.

Employment grade was based on three classes of civil service employment grades (Administrative / Professional or executive / Clerical or support) reported on a questionnaire. Information on ethnicity was collected at phases 1 and 5. A four-group classification (White / South Asian / Afro-Caribbean / Other) was derived from the phase 5 questionnaire, supplemented by the phase 1 questionnaire information (White / non-White) in case of missing.

Reference List

(1) Brunner EJ, Marmot MG, Nanchahal K, Shipley MJ, Stansfeld SA, Juneja M, Alberti KG. Social inequality in coronary risk: central obesity and the metabolic syndrome. Evidence from the Whitehall II study. *Diabetologia.* 1997;40:1341-1349.

(2) Carstensen M, Herder C, Kivimäki M, Jokela M, Roden M, Shipley MJ, Witte DR, Brunner EJ, Tabák AG. Accelerated increase in serum interleukin-1 receptor antagonist starts 6 years before diagnosis of type 2 diabetes: Whitehall II prospective cohort study. *Diabetes.* 2010;59:1222-1227.

(3) Brunner E, Stallone D, Juneja M, Bingham S, Marmot M. Dietary assessment in Whitehall II: comparison of 7 d diet diary and food-frequency questionnaire and validity against biomarkers. *Br J Nutr.* 2001;86:405-414.

(4) Miller MA, Kandala NB, Kumari M, Marmot MG, Cappuccio FP. Relationships between sleep duration and von Willebrand factor, factor VII, and fibrinogen: Whitehall II study. *Arterioscler Thromb Vasc Biol.* 2010;30:2032-2038.

(5) Gimeno D, Brunner EJ, Lowe GD, Rumley A, Marmot MG, Ferrie JE. Adult socioeconomic position, C-reactive protein and interleukin-6 in the Whitehall II prospective study. *Eur J Epidemiol.* 2007;22:675-683.

(6) Friedewald WT, Levy RI, Fredrickson DS. Estimation of the concentration of low-density lipoprotein cholesterol in plasma, without use of the preparative ultracentrifuge. *Clin Chem.* 1972;18:499-502.

(7) Armstrong NC, Paganga G, Brunner E, Miller NJ, Nanchahal K, Shipley M, Rice-Evans CA, Marmot MG, Diplock AT. Reference values for alpha-tocopherol and beta-carotene in the Whitehall II Study. *Free Radic Res.* 1997;27:207-219.

(8) Marmot M, Brunner E. Cohort Profile: the Whitehall II study. *Int J Epidemiol.* 2005;34:251-256.
