# Supplementary material for: Determinants of Aortic Stiffness: 16-Year Follow-Up of the Whitehall II Study
Source: PLoS One. 2012 May 22;7(5):e37165. doi: 10.1371/journal.pone.0037165 (PMC3358295; doi:10.1371/journal.pone.0037165)
Supplement: Table S1 — The fraction of the study participants with no information on the baseline determinant. (DOC) [file pone.0037165.s003.doc]

Table S1 The fraction of the study participants with no information on the baseline determinant

|  | **Men (%)** | **Women (%)** |
| --- | --- | --- |
| Age | 0 | 0 |
| BMI | 4 | 4 |
| Waist circumference | 5 | 4 |
| Hip circumference | 5 | 4 |
| Height | 4 | 4 |
| Diastolic blood pressure | 4 | 4 |
| Systolic blood pressure | 4 | 4 |
| Pulse pressure | 4 | 4 |
| Heart rate | 4 | 4 |
| Total cholesterol | 4 | 4 |
| HDL cholesterol | 5 | 4 |
| LDL cholesterol | 7 | 5 |
| Triglycerides | 5 | 4 |
| Apolipoprotein A-I | 4 | 4 |
| Apolipoprotein B | 4 | 4 |
| Lipoprotein (a) | 4 | 4 |
| Adiponectin | 53 | 57 |
| CRP | 10 | 12 |
| IL-6 | 11 | 12 |
| IL-1Ra | 53 | 56 |
| Fibrinogen | 12 | 12 |
| Von Willebrand factor | 14 | 17 |
| Factor VII | 11 | 12 |
| β-carotene | 26 | 21 |
| Alcohol intake | 3 | 2 |
| Vigorous exercise | 2 | 1 |
| Employment grade | 0 | 0 |
| Smoking habits | 4 | 4 |
| Fasting plasma glucose | 9 | 8 |
| 2-hour plasma glucose | 7 | 7 |
| Fasting serum insulin | 15 | 18 |
| 2-hour serum insulin | 8 | 7 |
| HOMA2-%B | 32 | 41 |
| HOMA2-IR | 32 | 41 |
| ISI_0,120_ | 16 | 19 |

BMI = body mass index; HDL = high density lipoprotein; LDL = low density lipoprotein; CRP = C-reactive protein; IL-6 = interleukin 6; IL-1Ra = interleukin 1 receptor antagonist; HOMA2-%B = β-cell function; HOMA2-IR = insulin resistance; ISI_0-120_ = insulin sensitivity index.
