# Supplementary material for: Determinants of Aortic Stiffness: 16-Year Follow-Up of the Whitehall II Study
Source: PLoS One. 2012 May 22;7(5):e37165. doi: 10.1371/journal.pone.0037165 (PMC3358295; doi:10.1371/journal.pone.0037165)
Supplement: Table S2 — Difference (95%-CI) in aortic pulse wave velocity at follow-up by a unit difference in determinants at follow-up. (DOC) [file pone.0037165.s004.doc]

Table S2 Difference (95%-CI) in aortic pulse wave velocity at follow-up by a unit difference in determinants at follow-up

|  |  | **Men** | **Women** |
| --- | --- | --- | --- |
| Anthropometrics | |  |  |
|  | Waist circumference (10 cm) | 0.51 (0.36;0.65)^‡^ | 0.36 (0.16;0.56)^†^ |
|  | Hip circumference (10 cm) | -0.12 (-0.3;0.06) | -0.35 (-0.59;-0.11)^†^ |
|  | Waist-hip ratio | 5.04 (3.70;6.39)^‡^ | 4.63 (2.79;6.47)^‡^ |
|  | Height (10 cm) | 0.05 (-0.05;0.15) | -0.02 (-0.19;0.15) |
| Hemodynamic marker | |  |  |
|  | Heart rate (10 bpm)|| | 0.41 (0.36;0.47)^‡^ | 0.23 (0.14;0.33)^‡^ |
| Lipids | |  |  |
|  | Total cholesterol (mmol/l) | 0.01 (-0.06;0.08) | 0.10 (-0.01;0.2)* |
|  | HDL cholesterol (mmol/l) | -0.26 (-0.43;-0.09)^†^ | -0.17 (-0.40;0.06)^†^ |
|  | LDL cholesterol (mmol/l) | -0.01 (-0.08;0.07) | 0.08 (-0.03;0.19) |
|  | Triglycerides (mmol/l) | 0.23 (0.14;0.31)^‡^ | 0.39 (0.20;0.58)^‡^ |
| Inflammatory markers | |  |  |
|  | Adiponectin (doubling) | -0.32 (-0.63;-0.01)* | -0.55 (-0.84;-0.26)^†^ |
|  | IL-1Ra (doubling) | 0.35 (-0.17;0.86) | 0.51 (0.06;0.97)* |
| Lifestyle | |  |  |
|  | Ex-smoker vs. never smoker | -0.06 (-0.19;0.08) | -0.11 (-0.33;0.10) |
|  | Current smoker vs. never smoker | 0.07 (-0.19;0.34) | 0.13 (-0.39;0.65) |
| Glucose metabolism | |  |  |
|  | Fasting plasma glucose (mmol/l) | 0.14 (0.07;0.20)^‡^ | 0.05 (-0.07;0.17)* |
|  | 2-hour plasma glucose (mmol/l) | 0.07 (0.03;0.10)^†^ | 0.10 (0.04;0.16)^†^ |
|  | HOMA2-%B (100 units)^§^ | 0.31 (0.08;0.53)^†^ | 0.84 (0.42;1.26)^‡^ |
|  | HOMA2-IR | 0.39 (0.25;0.52)^‡^ | 0.50 (0.25;0.74)^‡^ |
|  | ISI_0,120_ (100 units) | -1.02 (-1.52;-0.51)^‡^ | -1.29 (-2.11;-0.47)^†^ |

Analyses were adjusted for age, quadratic age, body mass index, mean arterial pressure at the time of aortic pulse wave velocity measurement, relevant treatment and events (heart rate: anti-hypertensive treatment and coronary heart disease events; lipids: lipid-lowering treatment; glucose metabolism: diabetes incidence).

*P<0.05, †P>0.01, ‡P<0.0001 for significance of the determinant

§P<0.05, ||P<0.01 for sex difference in the determinant

HDL = high density lipoprotein; LDL = low density lipoprotein; IL-1Ra = interleukin 1 receptor antagonist; HOMA2-%B = β-cell function; HOMA2-IR = insulin resistance; ISI_0-120_ = insulin sensitivity index.
