# Supplementary material for: Determinants of Aortic Stiffness: 16-Year Follow-Up of the Whitehall II Study
Source: PLoS One. 2012 May 22;7(5):e37165. doi: 10.1371/journal.pone.0037165 (PMC3358295; doi:10.1371/journal.pone.0037165)
Supplement: Table S3 — Baseline characteristics by follow-up participation and aortic pulse wave velocity measurement for those alive at follow-up. (DOC) [file pone.0037165.s005.doc]

Table S3 Baseline characteristics by follow-up participation and aortic pulse wave velocity measurement for those alive at follow-up

|  | **Follow-up and**  **aPWV** | **Follow-up and**  **no aPWV** | **Lost to follow-up** |
| --- | --- | --- | --- |
| N | 3,769 | 2,011 | 1,234 |
| Males | 75.8 (74.4;77.2) | 64.6 (62.5;66.7) | 66.5 (63.8;69.2) |
| Age (years) | 48.5 (5.8) | 50.3 (6.1) | 50.4 (6.4) |
| BMI (kg/m^2^) | 24.8 (3.2) | 25.8 (4.2) | 25.6 (3.9) |
| Waist circumference (cm) | 84.0 (10.8) | 85.7 (12.3) | 86.1 (12.4) |
| Hip circumference (cm) | 96.4 (6.5) | 97.9 (8.0) | 97.3 (7.6) |
| Waist to hip ratio | 0.87 (0.08) | 0.87 (0.09) | 0.88 (0.09) |
| Height (cm) | 173.6 (8.7) | 172.0 (9.5) | 171.3 (9.5) |
| Diastolic BP (mmHg) | 79.0 (9.1) | 80.1 (9.5) | 80.4 (10.0) |
| Systolic BP (mmHg) | 119.4 (12.9) | 121.3 (13.6) | 121.8 (14.8) |
| Pulse pressure (mmHg) | 40.3 (8.4) | 41.2 (9.2) | 41.5 (9.5) |
| Heart rate (bpm) | 63.7 (10.4) | 65.1 (11.1) | 66.5 (11.1) |
| Total cholesterol (mmol/l) | 6.4 (1.1) | 6.6 (1.2) | 6.6 (1.2) |
| HDL cholesterol (mmol(l) | 1.4 (0.4) | 1.4 (0.4) | 1.4 (0.4) |
| LDL cholesterol (mmol/l) | 4.3 (1.0) | 4.4 (1.0) | 4.5 (1.1) |
| Triglycerides (mmol(l) | 1.4 (0.9) | 1.5 (1.0) | 1.6 (1.0) |
| Apolipoprotein A-I (mg/dl) | 2.1 (0.3) | 2.1 (0.4) | 2.1 (0.4) |
| Apolipoprotein B (mg/dl) | 1.3 (0.3) | 1.3 (0.3) | 1.3 (0.3) |
| Lipoprotein (a) (mg/dl) | 30.2 (29.1) | 33.0 (31.3) | 35.6 (33.3) |
| Adiponectin (μg/ml) | 8.7 (6.5;11.9) | 9.3 (6.6;13.0) | 9.9 (6.6;14.2) |
| CRP (mgl/l) | 0.7 (0.4;1.5) | 0.9 (0.5;1.9) | 1.1 (0.5;2.3) |
| IL-6 (pg/ml) | 1.3 (0.9;1.8) | 1.4 (1;2) | 1.5 (1.1;2.3) |
| IL-1Ra (pg/ml) | 238.6 (189.2;308.3) | 257.4 (203.5;335.9) | 265.3 (194.9;345.6) |
| Fibrinogen (g/l) | 2.3 (0.5) | 2.4 (0.6) | 2.5 (0.6) |
| Von Willebrand's factor (IU/dl) | 102.2 (36.2) | 106.4 (38.8) | 111.0 (41.2) |
| Factor VII (% standard) | 87.8 (22.0) | 90.4 (22.3) | 90.2 (23.4) |
| Beta carotene (μmol/l) | 0.9 (0.5) | 0.9 (0.5) | 0.9 (0.5) |
| Alcohol intake (units/week) | 11.2 (12.6) | 10.4 (12.8) | 11.0 (14.6) |
| Vigorous exercise (hrs/week) | 0.9 (1.5) | 0.8 (1.5) | 0.7 (1.5) |
| Employment grade (%) |  |  |  |
| Administrative | 38.4 (36.8;40.0) | 33.8 (31.7;35.9) | 24.9 (22.5;27.4) |
| Prof/exec | 52.1 (50.4;53.7) | 47.5 (45.3;49.7) | 49.6 (46.8;52.4) |
| Clerical/support | 9.6 (8.6;10.5) | 18.7 (17.0;20.5) | 25.5 (23.1;28.1) |
| Smoking habits (%) |  |  |  |
| Never-smoker | 51.4 (49.8;53.1) | 44.4 (42.2;46.6) | 35.4 (32.7;38.2) |
| Ex-smoker | 35.2 (33.7;36.7) | 34.9 (32.8;37.0) | 30.0 (27.4;32.6) |
| Current smoker | 9.5 (8.6;10.5) | 14.1 (12.6;15.7) | 22.5 (20.2;25) |
| FPG (mmol/) | 5.2 (0.5) | 5.2 (0.7) | 5.3 (0.9) |
| 2hPG (mmol/l) | 5.5 (1.7) | 5.6 (1.9) | 5.6 (2.1) |
| HOMA2-%B | 78.8 (28.4) | 82.7 (30.5) | 83.2 (31.6) |
| HOMA2-IR | 0.91 (0.49) | 1.01 (0.59) | 1.02 (0.56) |
| ISI_0,120_ | 44.2 (18.6) | 42.7 (19.2) | 42.2 (18.3) |

Data are means (SD), medians (interquartile range) or proportions (95% CI) except for the number of participants (N).

aPWV = aortic pulse wave velocity; BMI = body mass index; BP=blood pressure; HDL = high density lipoprotein; LDL = low density lipoprotein; CRP = C-reactive protein; IL-6 = interleukin 6; IL-1Ra = interleukin 1 receptor antagonist; FPG=fasting plasma glucose; 2hPG=2-hour plasma glucose; HOMA2-%B = β-cell function; HOMA2-IR = insulin resistance; ISI_0-120_ = insulin sensitivity index.
